# Early tone categorization in absolute pitch musicians is subserved by the right-sided perisylvian brain

**DOI:** 10.1038/s41598-018-38273-0

**Published:** 2019-02-05

**Authors:** Anja Burkhard, Stefan Elmer, Lutz Jäncke

**Affiliations:** 1 Division Neuropsychology, Institute of Psychology, University of Zurich, 8051 Zurich, Switzerland; 2 University Research Priority Program (URPP), Dynamics of Healthy Aging, University of Zurich, 8051 Zurich, Switzerland

## Abstract

Absolute pitch (AP) is defined as the ability to identify and label tones without reference to keyality. In this context, the main question is whether early or late processing stages are responsible for this ability. We investigated the electrophysiological responses to tones in AP and relative pitch (RP) possessors while participants listened attentively to sine tones. Since event-related potentials are particularly suited for tracking tone encoding (N100 and P200), categorization (N200), and mnemonic functions (N400), we hypothesized that differences in early pitch processing stages would be reflected by increased N100 and P200-related areas in AP musicians. Otherwise, differences in later cognitive stages of tone processing should be mirrored by increased N200 and/or N400 areas in AP musicians. AP possessors exhibited larger N100 areas and a tendency towards enhanced P200 areas. Furthermore, the sources of these components were estimated and statistically compared between the two groups for a set of a priori defined regions of interest. AP musicians demonstrated increased N100-related current densities in the right superior temporal sulcus, middle temporal gyrus, and Heschl’s gyrus. Results are interpreted as indicating that early between-group differences in right-sided perisylvian brain regions might reflect auditory tone categorization rather than labelling mechanisms.

## Introduction

Absolute pitch (AP) musicians are able to identify tones without relying on an external or internal reference tone. AP is a rare ability for which some authors estimate the occurrence of 1 in 10,000 people^1^. However, recent large-scale studies have shown that the incidence of AP ability must be much higher particularly in professional musicians and is influenced by several factors (e.g., age of commencement, ethnicity, tonal language background, and anatomical prerequisites^2,3^). The neural and cognitive underpinnings of AP are still a matter of debate. For example, it remains unclear whether this ability originates from hereditary transmission, early exposure to environmental factors, or both (for a summary^4^). Another debate is concentrated around the question of which processes account for this very specific ability. An absolute pitch possessor (APP) is able to categorize, label, and produce a specific tone without making use of a reference tone^5,6^, while a relative pitch possessor (RPP) relies on the latter. AP is especially useful in categorizing the chroma of a given tone and not necessarily its height^1,5,6^. Thus, APPs are thought to experience pitches more categorically than RPPs or non-musicians. However, it is still a matter of debate whether AP describes an all-or-nothing or rather a gradually distributed ability. Already Bachem^7^ introduced the term quasi-AP, which describes the ability to estimate a given tone based on a single internal reference tone that is available. This internal reference might derive either from the deepest tone that a musician is able to sing or, for example, from the tuning tone for a given instrument. Based on such an internal reference, tone intervals can be calculated and the target tone can be estimated accordingly. Furthermore, Wengenroth *et al*.^8^ reported that a partial AP ability in musicians is more frequent than a perfect AP or no AP ability. This leads to the difficulty to fully disentangle the influences of these two abilities, possibly resulting in an overestimation of the AP ability. However, a consistent question in AP research is whether early perceptual stages of tone processing account for this ability, or whether later stages are the important ones (or both). The early perceptual encoding stages were suggested to be important by several previous studies^8–10^. In particular, Schulze *et al*.^9^ proposed that the difference between APPs and non-APPs lies in a specific perceptual ability of AP musicians that relies on the encoding of tonal information according to predefined pitch chroma categories. Otherwise, the labelling process is assumed to be a second component that is based on learned associations. In this context, Levitin^11^ proposed a two-component AP model where the ability of AP can be explained by pitch-memory and pitch-labelling. Pitch-memory is thought to be widespread even in non-APPs, whereas pitch-labelling is considered to be the main component accounting for AP ability^11,12^.

Due to the high temporal resolution of EEG or MEG, auditory evoked potentials represent an ideal biomarker for examining both early and late processes. The so-called early auditory evoked potentials (N100, and P200) primarily reflect sensory and perceptual processes at the initial stages of auditory analysis^13^ and are thought to be generated by the auditory cortex^14,15^. Furthermore, several studies were able to associate the N100/P200 complex with improved perception^16–20^. By contrast, later components occurring between 200–1,500 ms after stimulus onset have rather been associated with endogenous and cognitive processes^21^. In this vein, auditory categorization^22^, contextual influences, and lexical selection^23,24^ have been associated with N200 responses, while the N400 component can be used as a marker for semantic categorization^23^, episodic memory functions^25^ as well as pitch labelling processes^14^.

In a previous electrophysiological study conducted with AP and non-AP musicians, Wu *et al*.^26^ examined the N100 component under three different listening conditions. In one condition, participants had to listen passively to sine tones, in a second condition they made relative pitch (RP) judgments, and in a third condition, they had to label tones without using a reference. Even though the authors revealed global field power differences between the different conditions during the time window of the N100 component, they did not observe between-group differences. However, the intracortical sources of the N100 component were spatially more extended in the left and right auditory regions in the AP group during the labelling condition. Other studies that focused on the N100 component and their intracortical sources^27^ reported bilateral increased N100 responses in the auditory cortices among APPs during a labelling task, whereas the N100 component only increased in the left hemisphere for RPPs. However, it is still a matter of debate whether auditory cortex activity is differentially modulated within the AP group as a function of attention. Hirose and colleagues^27^ postulated that the right hemisphere is involved in pitch height analysis, while the left hemisphere is involved in label assignment. Furthermore, Pantev *et al*.^28^ found no differences in the N100 component between AP and non-AP musicians during a passive listening task, even though only the left hemisphere was examined. However, when they compared musicians against non-musicians, they observed stronger dipole moments for piano tones in the musician group. Furthermore, Itoh *et al*.^29^ suggested the existence of a so-called AP negativity, which occurred around 150 ms after stimulus onset in high-scoring APPs only. This AP negativity was observed during listening, pitch-naming, and auditory Stroop tasks. Furthermore, the amplitude of the AP negativity was modulated by stimulus congruence in the auditory Stroop task. Otherwise, differences in the P200 component have been observed during auditory feedback^30^ as well as during passive listening^8^ tasks with APPs showing larger amplitudes. Finally, Elmer and colleagues^14^ found no group differences between APPs and RPPs during a passive listening task for the N100 and P200 components. However, the authors reported increased N400 amplitudes for APPs during pitch-label association tasks.

While the aforementioned studies used EEG or MEG, other studies examined the functional anatomy of AP by means of fMRI and PET. When listening to tones compared to noise bursts, APPs revealed stronger blood flow in the left posterior dorsolateral frontal cortex^31^, leading the authors to assume that this region is involved in verbal-tonal association learning. Furthermore, when comparing an interval judgment task with noise bursts, APPs demonstrated stronger blood flow in the right middle and inferior temporal cortex, which are regions suggested to facilitate multi-modal processing^31^ and auditory object recognition^32^. The left dorsolateral prefrontal cortex was also associated with AP ability in a study by Ohnishi *et al*.^33^, who reported correlations between AP performance and hemodynamic responses in the left dorsolateral prefrontal cortex and planum temporale. Furthermore, stronger hemodynamic responses in the middle part of the left superior temporal sulcus (STS) were also observed in APPs during a pitch memory task^9^. In contrast, RPPs demonstrated stronger hemodynamic responses in the right superior parietal lobe and intraparietal sulcus. In general, the right auditory areas are thought to be more strongly involved in spectral analyses^27,34^, while the left auditory areas are suggested to be more strongly involved in rapid temporal processing^34^ and the assignment of tone labels^27^.

In this study, we examined the effects of attentive tone listening on classical ERPs, which have often been used to study auditory functions and related cognitive functions. In a second step, we explored the ERP-related intracortical sources and statistically compared them between the two groups by selecting a priori defined regions of interest (ROIs). This approach is particularly fruitful, especially when the data are collected using a high-density EEG array of 128 channels. Since we were interested in tracking the neural underpinnings underlying AP ability and not in differences between musicians and non-musicians, we compared musicians with AP to musicians without AP. A further innovative aspect of our study is that we measured a relatively large sample of 103 participants, whereas prior AP studies often used small sample sizes which makes it difficult to detect a true effect and increases the likelihood of reporting false-positive findings. Furthermore, since the labelling process is suggested to occur automatically in APPs^12^ an attentive listening task was used instead of a labelling task. In this context, participants were not forced to explicitly label tones, enabling a listening experience that is as natural as possible. Moreover, we used sine tones that were either in tune or slightly mistuned in order to prevent supportive instrumental cues.

In this EEG experiment, we addressed two different research questions. Firstly, we wanted to determine the time windows reflecting between-group differences, as this reveals the rough nature of the underlying perceptual and cognitive processes. In fact, early differences in the N100 and P200 components indicate an AP-related perceptual tone processing mode at the level of the auditory cortex. Based on previous studies, we expected higher N100/P200 amplitudes for the AP group compared to the RP group^8,10,30,35^. Furthermore, we assumed that putative between-group differences in the N200 and N400 components would reflect a differential engagement of cognitive processes such as lexical selection, memory functions, and tone-label associations (see for example^14^). Secondly, we expected that only APPs would be aware of the slightly mistuned tones since it has often been reported that APPs react disturbed and annoyed when recognizing mistuned acoustic information^12^. Therefore, we expected differential neuronal responses for APPs while listening to mistuned tones.

## Materials and Methods

### Subjects

In the present study, we measured 103 musicians with and without AP. Nine participants had to be excluded due to drug abuse and/or psychiatric disorders (depression and anxiety). All participants were tested with pure-tone audiometry (MAICO Diagnostic GmBh, Berlin) in the frequency range of 250–8,000 Hz. According to this procedure, all participants demonstrated a normal and non-pathological audiological status (i.e., all tested frequencies could be heard below a threshold of 30 dB). All participants gave informed consent and were paid for participation. The study was approved by the Ethics Commission of the University of Zurich in accordance with the declaration of Helsinki. All participants were highly trained musicians with a comparable proficiency level to avoid confounding factors regarding musical experience. To prevent an arbitrary classification criterion based on the AP-test score (see below) participants were divided into two groups based on self-report. The 49 participants who claimed to possess AP were grouped into the AP group and the remaining 45 musicians were assigned to the RP group. In the AP group, we examined eight wind players, 29 string players, one percussionist, nine pianists, and two singers. In the RP group, we included twelve wind players, 19 string players, five percussionists, seven pianists, and two singers. As depicted in Table 1, groups were comparable in gender, handedness^36,37^, the occurrence of synaesthesia, and bilingualism. Furthermore, musical aptitude was evaluated using the AMMA test^38^. Applying an additional in-house test, participants had to name ten intervals of two sequential sine tones of different frequencies to evaluate RP ability. This short procedure was applied as a screening test to confirm that all participants were characterized by RP abilities. General cognitive ability was evaluated using a standard German IQ screening test (KAI: Kurztest für allgemeine Intelligenz^39^). The age at which participants started to play an instrument, as well as the estimated training hours accumulated over the lifespan, were surveyed (Table 2).

**Table 1:** Gender (masculine/feminine), handedness (right/left/both), occurrence of synaesthesia (yes/no) and bilingualism (yes/no) proportions for both groups (AP/RP) are displayed.

	Gender	Handedness	Synaesthesia	Bilingualism
	(m/f)	(r/l/b)	(y/n)	(y/n)
**AP**				
N = 49	26/23	42/4/3	13/36	10/39
**RP**				
N = 45	20/25	40/4/1	13/32	15/30

**Table 2:** Means, standard deviations, and p-values (t-test, two-tailed) depicted for age, musical aptitude, correctly identified intervals, cognitive capability, age of commencement of instrumental training, and cumulative number of training hours across the lifespan.

	Age	AMMA	Interval	KAI	Age of Commencement	Training Hours
**AP**						
N = 49	27.43 (4.85)	66.37 (6.05)	7.71 (1.59)	122.74 (33.24)	6.02 (2.34)	15,445 (12,248)
**RP**						
N = 45	26.16 (4.58)	64.11 (6.66)	7.60 (2.03)	130.65 (29.47)	6.49 (2.51)	13,359 (9,705)
p-value	0.195	0.088	0.761	0.230	0.352	0.365

### AP Test

**Figure 1:**
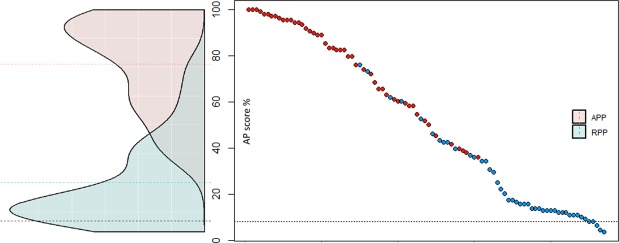
Distribution of AP scores (red: AP group, blue: RP group). The black dashed line represents the chance level (8.3%). On the left side, the distributions of APP and RPP are displayed with the corresponding dashed lines representing the means. On the right side, AP scores of all participants are depicted separately.

In order to verify AP ability, pitch labelling accuracy was also tested by a modified version of a previously used in-house developed test^40^ that each participant completed at home. With this procedure, we could assure that participants who claimed to possess AP had a corresponding score. The test consisted of 108 pseudo-randomly presented pure sine wave tones ranging from C3 to B5 (A4 = 440 Hz). After the presentation of each tone, the participants had to click on the appropriate label presented on a screen. Each tone trial had a time limit of 15 seconds. Immediately before and after each tone, two seconds of Brownian noise was presented to prevent mnemonic cues. If the participants did not select the corresponding tones in the time window of 15 seconds, the test continued with the next trial. After participants had accomplished the AP test they received an invitation for the EEG measurement. AP scores were obtained by computing the percentage of correct answers in the AP test (Fig. 1). An answer was classified as correct if the exact pitch chroma was identified. Octave errors were neglected because APPs are usually able to identify the chroma but not necessarily the height of a tone^1,5,6^. Afterward, a t-test (two-sided) was computed to uncover group differences. AP test performance substantially differed (as expected) between both groups (p < 0.001, Cohen’s d = 2.66, mean ± sd for the AP group: 76.43 ± 20.15, RP group: 24.91 ± 18.53). The performance of the RP group differed from chance level of 8.3% (p < 0.001, Cohen’s d = 0.90).

### Task

**Figure 2:**
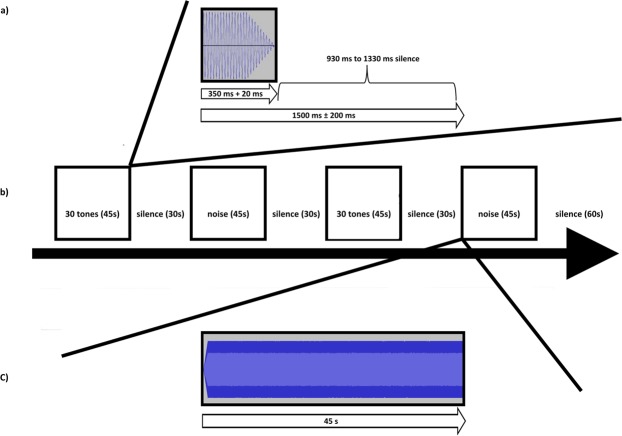
Experimental design. (**a**) Representation of a single tone trial. (**b**) Sequence of a whole presentation unit. (**c**) Continuous noise block.

The EEG experiment consisted of four blocks (Fig. 2) each of them lasting 45 seconds. During two blocks 30 tuned and 30 mistuned tones were randomly presented. Alternating to the tone blocks, two additional blocks with different continuous noise stimuli were presented for 45 seconds. These noise blocks were used to mask the memory trace between the tone blocks. We used two different noise stimuli to avoid boredom and habituation. Furthermore, the four blocks formed a presentation unit that was repeated four times during the experiment. The order of the blocks was randomized but each presentation unit always started with a tone block. Furthermore, after each block an interval of 30 seconds silence was introduced with longer silence (one minute) at the end of each presentation unit in order to provide the participants the possibility to have a rest. During the task, participants were instructed to attentively listen to the auditory stimuli. Thereby, we explicitly refrained from asking the participants to label the tones or to do some other cognitive tasks. The reason was that we wanted to keep tone presentation as natural as possible. In fact, when musicians hear tones they are normally not required to accomplish a given task. In addition, when RP musicians are instructed to label tones although they are not able to do that, they might use specific cognitive strategies. These strategies might be reflected in idiosyncratic neural activations that might hamper the detection of typical neural activations. During the whole procedure, participants were instructed to look at a fixation cross presented in the middle of the screen. Additionally, three minutes eyes open resting state was recorded.

### Stimuli

The auditory stimuli consisted of sine tones of different frequencies that were randomly presented, ranging from C4 to B4. Tones were either in tune or slightly mistuned. The mistuned tones were either a quarter halftone flat or sharp respective to the in tune ones. Tone blocks comprised randomly presented tones of different frequencies with a duration of 370 ms (linear fade-out = 20 ms). The tones were presented with an inter-trial interval of 1,500 ms and a latency jitter of ±200 ms resulting in an inter-stimulus interval in the range of 930–1,130 ms. Additionally, two noise stimuli (Brownian and white) were randomly presented, whereby each kind of noise was presented once in each presentation unit. The noise stimuli were faded in for 20 ms and were continuously presented during 45 seconds. All stimuli were generated and processed with Audacity (Version 1.3 Beta, The Audacity Team, USA).

### EEG recording and pre-processing

EEG was acquired using HydroCel Geodesic Sensor Net with 128 Ag/AgCl electrodes, a Net Amp 400 amplifier system, and Net Station acquisition software (Version 5.2.0.2) manufactured by Electrical Geodesic (Electrical Geodesics Inc., Eugene, Oregon, USA). Impedances were kept below 25 kΩ using a potassium-chloride solution. The EEG signal was online-filtered from 0.01 to 100 Hz with an analogue bandpass filter and digitized at a sampling frequency of 1,000 Hz. The vertex electrode Cz served as an online reference. Auditory stimuli were presented binaurally at a sound pressure level of 75 dB using Bose Companion 2 Series III external speakers (Bose Corporation, Framingham, Massachusetts, USA). Participants were asked to relax during the entire procedure to avoid muscle artefacts. Furthermore, prior to the EEG measurement, participants were instructed about the consequences of eye blinks and saccades.

EEG recordings were converted into Brain Vision Analyzer format using EEGLAB^41^. Pre-processing was done with Brain Vision Analyzer (Version 2.1, Brainproducts, Germany). As a first step, the outer ring of electrodes was removed due to potential contamination of muscle artefacts (108 electrode sites remained). The data were offline-filtered from 0.1–30 Hz with an infinite impulse response filter. A semi-automatic individual independent component analysis was performed to remove eye movement artefacts^42–44^. Afterward, the average reference was calculated and the vertex electrode was re-used as an active electrode. Additionally, an automatic raw-data inspection was performed to exclude segments with excessive amplitudes. Thresholds were set as follows: 50 µV/ms maximal allowed voltage steps between two sample points, 100 µV maximal allowed absolute difference during a time window of 200 ms, ± 100 µV maximal/minimal allowed amplitude and 0.5 µV lowest allowed activity during an interval length of 100 ms.

### ERP analyses

**Figure 3:**
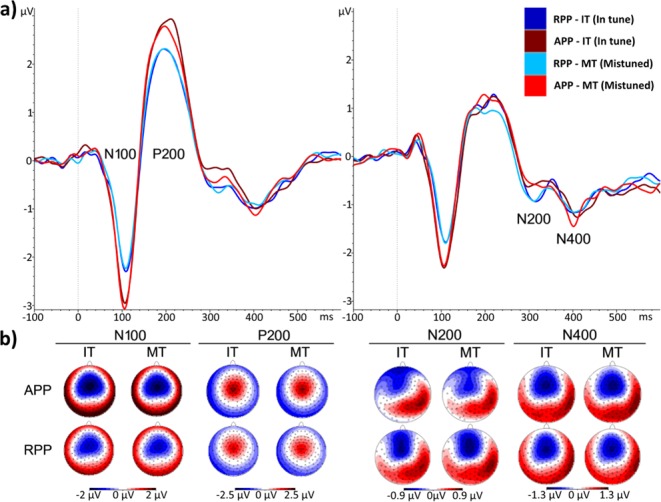
ERPs and topographic scalp maps for the different components of interest. ERPs for the APPs are coloured in red and those for the RPPs in blue. Darker colours represent the IT condition and lighter colours the MT condition. (**a**) On the left, ERPs for the N100 and P200 components for electrode site Cz are shown. N200 and N400 at electrode site Fz are depicted on the right. (**b**) Topographic scalp maps for the components of interest and the different conditions (IT, MT) with the corresponding µV scales.

The EEG data related to the tones were segmented corresponding to the different conditions, namely in tune (IT) and mistuned (MT). A baseline correction of −100 ms to stimulus onset was performed. Afterward, data were averaged for each participant and condition, exported, and further analysed with an in-house Matlab script (Version R2015b, Mathworks, USA). This script extracted the signed area^45^ over a predefined time window. For the negative components (N100, N200, N400) the signed area underneath the baseline and for the positive component (P200) the signed area above the baseline was exported. One of the main advantages of using the signed area is that fairly large time windows can be used without voltage cancelation and with less bias towards choosing arbitrary time windows. Furthermore, the ERP components are treated as signals that extend over time instead of focusing on the peak voltage or on small time windows. Grand averages were computed separately for both groups (AP, RP) and conditions (IT, MT) (Fig. 3). Furthermore, electrode sites for statistical analyses were selected based on the according to voltage maxima in the grand averages. For the N100 component, signed areas were extracted during a time window of 50–200 ms, whereas for the P200 component area was evaluated in the range of 120–400 ms^46^. Both components were exported for the Cz electrode site according to the maxima shown in Fig. 3. Signed area values corresponding to the N200 and N400 components were extracted in time windows of 270–350 and 350–470 ms at electrode Fz according to the maxima.

### Source reconstruction

For all ERP components that revealed differences in the conventional ERP analyses described above, intracortical sources were computed using the Brainstorm toolbox^47^. Based on the fact that we revealed between-group differences that were independent of the experimental conditions (IT, MT), brain responses to all tones were used for source reconstruction. For source reconstruction in each participant, we used the average files containing all tones. Noise covariance matrices were computed based on individual resting states. Open MEEG software^48,49^ was used to construct head models with 15,002 dipoles (1 × 1 × 1 mm surface grid). This software reconstructs a forward model that best fits brain activity measured at the sensor level. Electrode positions were defined by a standard EGI template provided by Brainstorm. The default anatomy template of the International Consortium for Brain Mapping (ICBM-152) provided by Brainstorm was used. Conductivity values (scalp 0.44, skull 0.018, and brain 0.25) were chosen based on the recommendations from Song *et al*.^50^. Inverse solutions were computed by using minimum norm imaging to calculate current density maps with constrained source orientations. Based on the voltage measured at the sensor level, inverse solutions estimate the corresponding distributions of active cortical sources.

**Figure 4:**
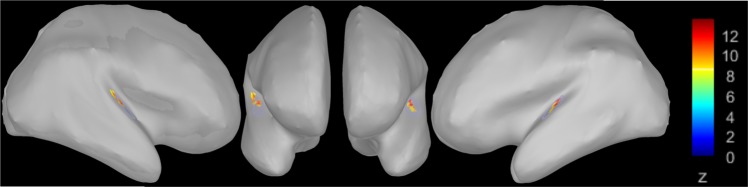
Source estimation for the peak amplitudes of the N100 component at 107 ms. Centroid voxels are located in the bilateral Heschl’s gyrus (MNI coordinates for the left hemisphere: x = −42, y = −23, z = 13; right: x = 43, y = −24, z = 13). Spatial smoothing was applied for visualization.

Before starting with source reconstructions of the single ERP components, the reconstruction of the N100 source (peak) was used as a localizer in order to provide evidence for a meaningful inverse solution. Based on the fact that it has frequently been shown that the main generators of the N100 component are located in the primary and secondary auditory cortex^51–54^, we tested whether this was also true for our data. Figure 4 shows, that this was indeed the case.

**Figure 5:**
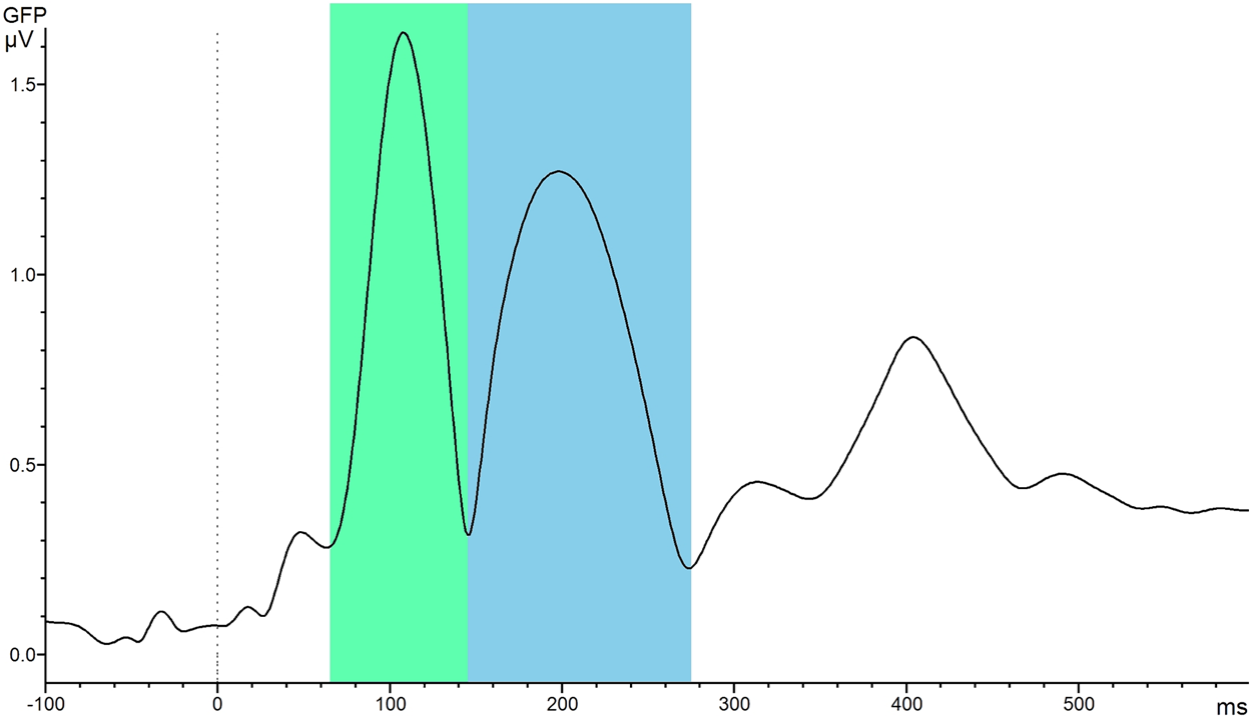
Time windows used for source reconstruction based on the global field power for the N100 (70–145 ms, green) and the P200 (145–275 ms, blue) components.

Time windows for statistical source analyses of the ERP components were chosen based on the global field power of the grand averages which were computed with Brain Vision Analyzer (Fig. 5). Using the same time windows as for the ERP analyses (i.e., signed area) was not an option because the time windows for the signed area were fairly wide. If the same time windows would have been taken for source reconstruction, the estimation would be contaminated with other neighbour components. To get a good temporal approximation of the single components, global field power was used instead^55^. Therefore, a grand average based on the segments of all tones was used for the determination of the time windows for source analysis (Fig. 5). Time windows used for source reconstructions were 70–145 ms for the N100 and 145–275 ms for the P200 component.

**Figure 6:**
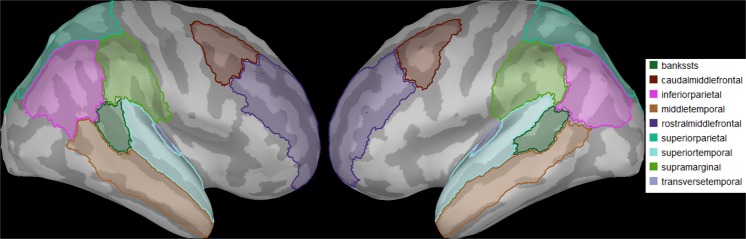
Nine ROIs were selected in each hemisphere. The names of the ROIs are depicted on the right with the corresponding colours.

At the single-subject level, z-score normalizations were performed relative to baseline (−100 ms to stimulus onset) to make amplitudes comparable between subjects. Furthermore, a priori defined ROIs containing temporal, frontal, and parietal areas were chosen based on previous literature on AP^4,8–10,14,31,33,56–60^. Figure 6 shows the 18 ROIs, which were selected based on the Desikan-Killany atlas^61^. There was one source signal for each vertex (e.g., voxel) contained in a specific ROI because constrained dipole orientations were used. Furthermore, grand averages of the source reconstructions for each group were calculated, as well as differences between the groups (AP-RP) to visualize which group had stronger/weaker activations.

### Statistical analysis

#### ERP

Four two-way (2 × 2) ANOVAs with repeated measurements on one factor (IT vs. MT) and one grouping factor (AP vs. RP) were computed with area values separately for each component of interest (N100, P200, N200, N400). Before computation, we tested whether the data were normally distributed. Statistical analyses were executed with SPSS (Version 22, IBM, USA). As dependent variables, we used signed area values measured at electrode Cz for the N100 and P200 components and at electrode Fz for the N200 and N400 waveforms.

Post-hoc bivariate Pearson correlations (one-tailed) were calculated between AP scores and signed area values for each ERP component in the whole sample of participants as well as separately for the two groups. Since the correlations served for descriptive purposes, no correction for multiple comparisons was applied.

#### Source reconstructions

FieldTrip source statistics^62^ were performed calculating independent t-tests (two-tailed) for the intracortical sources in the predefined ROIs for each ERP component that revealed a significant result in the previous analyses. In particular, we performed non-parametric cluster-based permutation statistics with an alpha set at p = 0.05. Here, samples exceeding a t-value associated with an alpha of 0.05 were clustered according to their spatial adjacency. Each cluster’s sum of the t-values was the basis for its cluster-level statistic, and group differences were tested with the maximum of those statistics. By setting the threshold for the cluster alpha to p = 0.05, a t-value thresholding at the 2.5th and the 97.5th quantiles was attained. Significance probability was calculated with the Monte Carlo method using 50,000 randomly selected permutations. A cluster-wise correction for multiple comparisons was applied.

Furthermore, bivariate Pearson correlations (one-sided) were computed for the whole sample and also separately for the two groups between AP scores and the current density values of the ROIs that revealed a significant group difference in the cluster-based permutation test. For each of these ROIs, activity was averaged over all vertices included in the corresponding ROI. Furthermore, an average over time was calculated for the duration of the whole component. This resulted in one value per ROI and participant.

## Results

### Results with respect to the amplitudes of the N100, P200, N200 and N400 components

**Figure 7:**
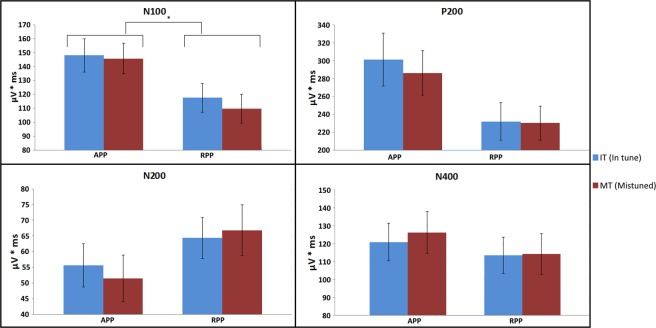
Signed area values for the components of interest (N100, P200, N200, and N400). Means and standard errors of means are displayed. Values for the AP group (APP) are shown on the left while those for the RP group (RPP) are displayed on the right. Furthermore, amplitudes for the in tune (IT) condition are blue coloured while those for the mistuned (MT) condition are red. The unit for the signed area is µV*ms.

As depicted in Figure 7, the evaluation of the N100 component revealed a main effect of group (F(1,92) = 4.801, p = 0.031, d = 0.45, CI_95%_ = 0.043–0.863). The AP group showed larger area values compared to the RP group, irrespective of the condition (IT, MT). No other main effect or interaction reached statistical significance. For the P200 component there was a tendency towards a significant group difference (F(1,92) = 3.574, p = 0.062, d = 0.391, CI_95%_ = 0.018–0.799) with APPs showing slightly larger signed areas in comparison to RPPs. For the N200, and N400 components we did not reveal significant results.

Weak but significant correlations (uncorrected) were only found for the P200 component. In particular, within the AP group we revealed a positive relationship between AP score and the MT-related area values (r = 0.24, p = 0.047). Otherwise, within the RP group, we found a negative correlation between AP score and P200 area (r = −0.28, p = 0.032; Table 3).

**Table 3:** Correlations between the AP scores and ERP area depicted for all components and conditions (IT: in tune, MT: mistuned). The correlation coefficient r and the p-value (uncorrected) are displayed for all participants (All), and also for the two groups (AP and RP group) separately. Significant correlations are highlighted with an asterisk.

	IT N100 MT		IT P200 MT		IT N200 MT		IT N400 MT	
All								
r	0.19	0.24	0.18	0.18	0.00	−0.08	0.11	0.15
p	0.032*	0.009*	0.045*	0.042*	0.486	0.210	0.150	0.079
AP group								
r	0.12	0.19	0.22	0.24	0.22	0.21	0.05	0.14
p	0.207	0.097	0.063	0.047*	0.063	0.079	0.358	0.165
RP group								
r	−0.00	−0.05	−0.28	−0.23	0.02	−0.12	0.19	0.15
p	0.495	0.365	0.032*	0.062	0.444	0.217	0.110	0.169

### Results with respect to the intracortical sources of the N100 and P200 components

**Figure 8:**
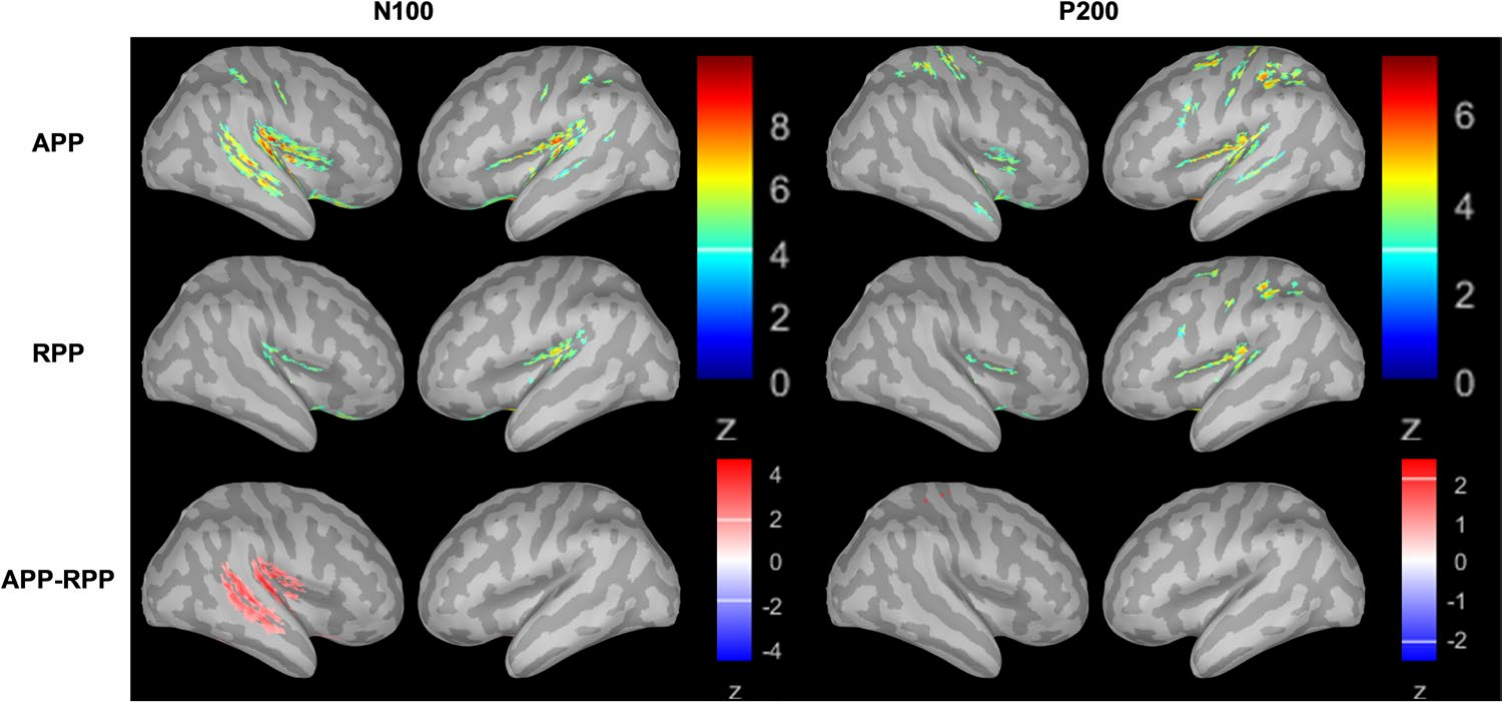
Source reconstructions in the AP and RP groups for the N100 and P200 components (z-transformed relative to the baseline). Source activations for the N100 are depicted on the left side, those for the P200 on the right side. The upper row shows the source activation for the AP group and the middle row for the RP group. In the bottom row, the differences between the source activations of the AP group and the RP group are displayed (AP group-RP group). Z-scores in the bottom row were thresholded at z = 2.1, which corresponds to a p-value of 0.01. Activations in the STS, MTG, and HG in the right hemisphere showed statistical differences between the two groups. Spatial smoothing was applied for visualization purposes.

Based on the fact that the ERP analyses revealed a main effect of group for the N100 component and a tendency towards significance for the P200 component, source reconstructions were only calculated for these two components. For the N100 component (70–145 ms) significant clusters between AP and RP groups were found in the right hemisphere (Fig. 8). Between-group differences were most pronounced in three ROIs, namely STS (cluster size = 68, p = 0.002), middle temporal gyrus (MTG) (cluster size = 58, p = 0.027), and the transverse temporal cortex, which corresponds to Heschl’s gyrus (HG) (cluster size = 72, p = 0.004). Since permutation statistics do not enable to determine the direction of the effects, we additionally computed between-group differences of the source activations (AP-RP) (Fig. 8). No significant result was revealed for the P200 component.

**Figure 9:**
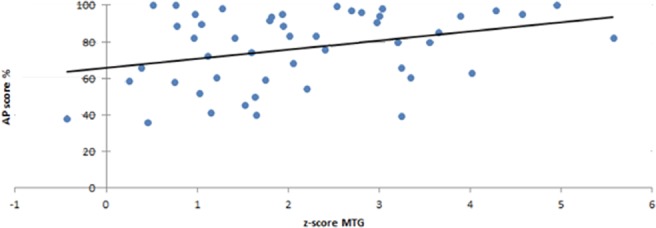
Correlation between the AP score and the z transformed activation in the middle temporal gyrus (MTG) for the AP group.

The results of the correlations between the AP scores and the ROIs showing group differences are displayed in Table 4. These additional analyses revealed a significant correlation for the right MTG in the AP group (r = 0.33, p = 0.010) (Fig. 9).

**Table 4:** Correlations between the AP scores and mean current density values in the right STS, MTG, and HG. Correlations are displayed for all participants (All) as well as for the AP and RP group separately. The correlation coefficient r and the p-value (uncorrected) are depicted. The asterisk depicts significance.

	STS	MTG	HG
All			
r	0.34	0.39	−0.31
p	<0.001*	<0.001*	0.001*
AP group			
r	0.16	0.33	0.12
p	0.133	0.010*	0.199
RP group			
r	−0.15	0.16	−0.01
p	0.156	0.149	0.477

## Discussion

### General discussion

In the present study, we examined the neural underpinnings of AP processing during attentive tone listening. We explicitly used attentive listening without an additional cognitive task (e.g., tone labelling or tone discrimination, etc.) in order to keep the listening condition as ecologically valid as possible. In normal music and auditory listening situations, musicians are not explicitly asked to solve a task related to the listening condition. They simply listen and apply the analysis strategies, which they have largely implicitly access to. In the case that AP musicians use specific auditory object analysis strategies automatically, we aim to identify the neural underpinnings of these strategies with this study. To achieve this, we used two different approaches: First, we analysed the N100, P200, N200, and N400 components to conform to earlier ERP studies on AP musicians. Our study was based on the so-called two-component model of AP processing^11^ as well as on studies assuming an early perceptual categorization (or perceptual memory) and a subsequent cognitive labelling process^14,57^. Differences in perceptual categorization between both groups should thus be reflected in differences in early ERP manifestations, namely the N100 and P200 components and their associated intracortical sources. In the case of significant differences for the subsequent labelling process, we anticipated significant differences for the late ERP components and their associated intracortical sources, namely the N200 and N400 components. Significant group differences were only identified for the N100 (and partially for the P200), with APPs exhibiting larger area values for both components. Second, we analysed the intracortical sources of the N100 component and revealed stronger current densities in APPs in the right STS, MTG, and HG. In addition, we observed a weak but significant correlation between activation in the MTG and AP performance for the AP group.

In our work, we also hypothesized that only AP musicians would recognize the slightly mistuned tones and therefore exhibit different neuronal responses compared to RPPs. We expected this difference in any component of interest (N100, P200, N200, and N400). This should be reflected by an interaction effect of the two-way repeated measurement ANOVAs performed with the area values. However, no such interaction effect was found. After the EEG experiment, participants completed a short questionnaire to evaluate whether the mistuned tones were identified as such. The question was whether the participants consciously noticed that some tones were mistuned (answer alternatives: yes/no). APPs (45 of 49), as well as RPPs (34 of 45), mentioned that they detected the mistuned tones. Furthermore, RPPs and APPs did not significantly differ in terms of musical experience. Therefore, it is possible that this could account for the missing interaction effects. In the following section, we will discuss how and whether our findings fit the existing literature.

### Discussion with respect to the ERP components

Studies examining differences between AP and RP musicians with respect to the N100 amplitude during tone listening paradigms are relatively rare^10,14,26–30,63,64^. Such studies have applied different paradigms (passive listening, attentive listening, tone labelling, tone discrimination, and vocal feedback discrimination), and examined different samples (children and adults) with mostly small sample sizes (7–22 AP musicians). Since these studies differed in many aspects, the reported results with respect to the N100 component are undoubtedly mixed. The majority of these studies have reported little or no differences between AP and RP musicians. One of the first studies of this type did not observe any differences in the magnetic counterpart of the N100 (N100m) component between APPs and RPPs^28^. In this study, participants had to listen to piano and pure tones while watching a cartoon. The authors, however, identified enlarged dipole moments in the auditory cortex in response to the piano tones in comparison to sine tones. This enlargement was generally present in musicians, with no differences between APPs and RPPs. In a follow-up study of the same research group, different activations for the N100m component between AP and non-musicians were reported^10^. The participants of this study had to listen to different stimuli while counting some of them to ensure attentional processing of the tones. Hirose *et al*.^27,63,64^ used tone identification and passive listening tasks and reported stronger N100m responses to tones among AP musicians. These stronger N100m amplitudes were also associated with generally stronger dipoles bilaterally in the auditory cortex. Further evidence for differences during early pitch processing stages comes from Itoh *et al*.^29^, who identified a negativity over left posterior-temporal electrode positions, an ERP component taking place around 150 ms after stimulus onset and which was only present in AP musicians performing excellently in an AP task (High-AP). This component (AP negativity) was present in both listening and pitch-labelling tasks. Furthermore, Wu *et al*.^26^ recorded conventional ERPs to auditory stimuli during three experimental conditions (inattentive listening to tones, tone labelling, and relative pitch labelling), and identified no between-group differences for all conditions. However, analysis of the intracortical sources of ERPs uncovered stronger and more widespread activations bilaterally in the auditory cortex as well as in the occipital and parietal areas, particularly during the tone labelling task. Another study from Elmer and colleagues^14^ using a passive listening paradigm reported no difference between AP and RP musicians with respect to the N100 amplitude. The most recent study^30^ using a vocal pitch error detection task uncovered a massively stronger N100 response to pitch-shifted voice feedback over right-sided electrode clusters for both AP and RP musicians compared to the non-musician group. The P200 component obtained over a left-sided electrode cluster was increased in AP musicians compared to RP musicians.

Taken together, the aforementioned studies have reported mixed results with respect to N100 or N100m amplitudes. Previous studies reported enlarged N100 amplitudes to musical stimuli during tone identification^10,27,30,63,64^. However, it is difficult to compare these studies due to their differences with respect to the paradigms used, samples, and analysis techniques. However, it cannot be ruled out that attention or other cognitive processes might have influenced the results since the paradigms require different levels of attention and stimulate different cognitive processes. This is particularly problematic for RP musicians, as some of the required tasks (e.g., labelling) are difficult or even impossible for them. However, when AP musicians automatically use a particular processing strategy, they should have used it particularly during attentive listening. In fact, we observed enlarged N100 components in AP musicians, a result which supports the notion that early auditory processing steps are different in AP musicians. Thus, AP musicians either allocate more attention to the tones or automatically categorize the tones at early processing steps. Nevertheless, we have to be careful not to over-interpret this finding since the effect size for this between-group differences was small to medium (Cohen’s d = 0.453) and most importantly much smaller than the effect size observed for the performance in the AP test (Cohen’s d = 2.66). Thus, the N100 amplitude did not reflect all neurophysiological processes involved in AP processing. However, the exact psychological functions responsible for this are difficult to determine. This problem will be explained in greater detail in the following section, which discusses the intracortical sources of the N100 component, as these results help us understand the neural underpinnings of AP processing.

### Discussion on the intracortical sources of the N100 component

The intracortical sources of the N100 component revealed substantial between-group differences with respect to current densities in the right-sided STS, MTG, and HG. These brain areas are part of the perisylvian brain, which is distinctly organized anatomically and functionally in AP musicians compared to RP musicians and non-musicians^8,9,33,40,58,65–72^. Based on previous studies, it is not entirely clear whether the right, left, or both perisylvian areas are differently involved in AP processing. Some anatomical studies reported specific anatomical features on the right side^8^, while others have identified a stronger relative leftward asymmetry^65^. The same pertains to neurophysiological activations obtained from fMRI, EEG, and MEG studies^9,29,33,71,73^. Based on the current status of knowledge, it is not easy to reconcile these different findings, since most of the studies (especially the functional studies) differ in terms of the applied task, statistical analyses, and sample size. Therefore, it remains challenging to use the available literature to reconcile the right-sided activation preponderance in the STS, MTG, and HG that occurs during the first 100 ms of auditory processing. Otherwise, the increased brain activity we revealed in APPs in the right HG, STS, and MTG might be reconcilable with previous models of auditory processing suggesting an advantage of right-sided perisylvian areas for spectral processing^74,75^. Furthermore, it is noteworthy to mention that the tones we used in our experiment had a duration of 370 ms, which roughly corresponds to a frequency of 3 Hz. Low-frequency oscillations have previously been shown to be more pronounced in the right compared to the left auditory cortex^76^, and to play an important role in packing the multi-time speech signal into units of the appropriate temporal granularity^77^.

Previous fMRI and PET studies on AP musicians have used pitch memory tasks^9,73^, auditory Stroop tasks^31,71^, pitch detection tasks^31,71^, or simply passive listening to a musical piece as stimulation^33^. In addition, prior studies have largely worked with small samples only including a few AP musicians (10–18). Thus, these studies can hardly be compared to our work, which explicitly used an attentive listening paradigm to keep the listening condition as ecologically valid as possible. This is important in that musicians are normally not asked to label, discriminate, or rehearse single tones in natural music listening conditions but simply play and listen to music. In addition, we included a large sample of 49 AP musicians in order to avoid false positive results. Nevertheless, besides the discrepancies observed in the results and paradigms used, some similarities exist between our findings and the results of the aforementioned studies.

In line with several previous studies, we assume that AP musicians (in contrast to RP musicians) automatically categorize tones during very early processing stages, even when they are not required to do so. In these early processing stages (approximately 100 ms after stimulus presentation), many bilateral perisylvian brain areas—particularly the HG, STG, MTG, and STS—are involved in a slight preponderance of the right-sided areas (see Fig. 8). Several studies have shown that the STS, MTG, and STG are brain areas that are involved in categorization and multimodal integration processes. For example, Klein and Zatorre^78^ showed larger right- and left-sided STS activity during categorical tone processing in non-AP subjects. Moreover, left-sided involvement of the STS has been reported by Schulze *et al*.^9,71^ in pitch memory and auditory Stroop tasks for AP musicians compared to RP musicians. They interpreted these findings as evidence for an early encoding process which is involved in the categorization of tonal information into pitch chroma classes and is most likely controlled by the STS. Activations in the MTG have previously been associated with multimodal processing^31^, lexical processing^40,79^, and access to stored pitch categories^68,69^. Therefore, it might be that the MTG is functionally stronger associated with tone categorization in AP musicians, while the right HG might generally play an important role in AP, as highlighted by Wengenroth *et al*.^8^. Additionally, Wengenroth and colleagues observed increased volumes of the HG in APPs, which was highly correlated with AP performance. Accordingly, the authors suggested that the right HG is a pivotal structure for AP perception, and stated that the left hemisphere is important for the labelling process.

In the present study, the preponderance of right-sided activation during the very early auditory processing stages could indicate a process specific to AP musicians. We assume that this specific process reflects automatic tone categorization that is supported by a network comprising several perisylvian brain areas. This network, which is lateralized to the right hemisphere, is different from what is known from studies examining neural activations in these brain areas during different attention conditions^80,81^. When attention is directed to both ears, hemodynamic responses increase bilaterally in the perisylvian brain, and not only in one hemisphere. Thus, we assume that the right-sided activation increase identified in the present study indicates an AP-specific neural activation, which is most likely related to automatic tone categorization.

In this context, we would like to emphasize that several EEG experiments have shown that brain activation can quickly change between the right and the left perisylvian brain, even during the first 1,000 ms of auditory information processing^16,82^. Thus, lateralized activations obtained from fMRI and EEG/MEG experiments are difficult or even impossible to compare since both techniques measure neurophysiological processes at different time scales. Finally, it is important to mention that according to our results we did not reveal functional evidence reflecting pitch labelling mechanisms. In fact, pitch labelling has more likely been associated with late EEG manifestations^14^ as well as with activation patterns in the language-dominant left hemisphere^26,31,83^. Accordingly, we propose that the increased current density values we revealed in the right hemisphere in AP compared to non-AP musicians reflected an optimization of auditory objects recognition or categorization processes rather than labelling per se^72^.

### Possible implications for AP perception and labelling

Our study indicates that AP musicians automatically activate a right-sided perisylvian brain network during attentive tone listening. This additional activation might be the neural underpinning of tone classification. However, it is also conceivable that AP musicians allocate more attention to the incoming tone stimuli, thereby resulting in a more widespread activation in the perisylvian brain. However, even though one may assume that attentional enhancement and tone categorization interact when AP musicians listen attentively to tones, attentional enhancement effects could not entirely explain this additional right-sided activation since attentional enhancement effects are mostly bilateral^80,84–86^. In addition, it is noteworthy to mention that we did not reveal between-group differences in the MT condition, leading to suggest that attention functions cannot substantially explain the increased right-sided activity we revealed during early stages of auditory processing in AP compared to non-AP participants.

Interestingly, we did not find between-group differences for later processing stages, which are thought to control tone labelling. This result is unexpected since tone labelling has been proposed to be the idiosyncratic trait of AP^12^. A possible explanation could be that the labelling process occurred very early in time and involved perisylvian brain areas. Otherwise, it is also possible that the labelling process itself is not fully automatic as hitherto assumed but only activated when requested. Finally, it is important to remark that previous authors suggested a contribution of the prefrontal cortex to labelling processes^31,33,87^. However, signal transmission from the auditory cortex to anterior brain sites needs time and is therefore expected to be reflected by other ERP components than the N100 response. This perspective is also compatible with the results of the source estimation we used showing that the N100 was associated with current densities in auditory-related brain areas, which have previously not explicitly been associated with labelling processes.

## Limitations and Outlook

A limitation which concerns AP studies, in general, is how to measure and objectify this ability. Many studies (as we did) rely on self-report for classifying musicians into AP and RP musicians. The reason for this procedure is that it is not entirely clear which performance cut-off should be used to decide whether the musicians are AP or RP musicians. A more objective and widely accepted measurement is urgently needed for this field (e.g. mean absolute deviation as proposed by Bermudez and Zatorre^88^) because the answers of the participants may also rely on self-esteem, social desirability, or other motivational reasons. Some people possess AP for a single tone and when asked to identify a given tone they can do it by calculating the interval between those two tones. This kind of quasi-AP^1^ is a strategy which needs more time than simply identifying a tone without a reference tone. Therefore, exact reaction time measurements could be useful for evaluating AP abilities^88^. In addition, future studies should try to develop more sensitive tests that enable a meticulous estimation of both AP and RP abilities. This implies that instead of merely improving the sensitivity of AP tests it would be advantageous to additionally implement a test that screens RP abilities. Furthermore, to investigate whether early and/or later processing stages are involved in determining AP ability, more studies that investigate basic auditory processing are needed. In this context, it is very important to also publish those studies with null effects. Although we explicitly conducted this study using attentive listening and not labelling, it will be interesting to more carefully examine the neurophysiological underpinnings of tone labelling, tone discrimination, and pitch memory in AP and RP musicians. Finally, one might argue that we should have used subgroups of AP musicians in order to examine whether different performance levels in the AP test might be related to the measured neurophysiological measurements. We refrained from subdividing the AP group into several subgroups in order to keep the AP group large enough for sustained enough statistical power. However, we performed several correlation analyses to examine whether the AP performance correlated with the amplitudes of the ERP components and the current densities values of perisylvian brain areas. These correlations were mostly small, indicating that the neurophysiological responses to attentive tone listening do not entirely depend on the performance in the standardized AP test.

### Conclusion

The present work included the largest number of AP and RP musicians used in any neuroscientific study on this topic to date and examined whether attentive tone listening evoked different neural responses in AP and RP musicians. We identified differences at the early stages (N100) of auditory processing between AP and RP musicians, which were accompanied by stronger activations in right-sided perisylvian brain areas comprising the STS, MTG, and the HG in APPs. In contrast, no differences were observed at the later stages of auditory processing. The data presented here suggest that differences between AP and RP musicians exist at early stages of auditory processing reflecting categorization, which is pivotal for AP musicians. This early auditory processing is controlled by bilateral perisylvian brain areas with a preponderance of the aforementioned right-sided perisylvian network.

## Data Availability

The raw data supporting the conclusions of this manuscript will be made available by the authors on reasonable request.
